# The effect of COVID-19 school closures on adolescent sleep duration: an uncontrolled before-after study

**DOI:** 10.1186/s12889-021-11589-9

**Published:** 2021-08-10

**Authors:** Qiguo Lian, Xiayun Zuo, Xiangyang Zhong, Xiaowen Tu, Jiashuai Zhang, Chang Shu, Chunyan Yu, Chaohua Lou

**Affiliations:** 1 NHC Key Lab. of Reproduction Regulation (Shanghai Institute for Biomedical and Pharmaceutical Technologies), Fudan University, 779 Laohumin Road, Shanghai, 200237 China; 2 Shanghai Jing’an Education College, Shanghai, 200070 China; 3 School of Public Health, Fudan University, Shanghai, 200032 China

**Keywords:** COVID-19, School closure, Sleep duration, Adolescent, China

## Abstract

**Background:**

The coronavirus disease 2019 (COVID-19) pandemic affected almost 1.6 billion students or more than 90% of learners globally. However, the effect of school closures during COVID-19 pandemic on adolescent sleep duration remains unclear.

**Methods:**

We undertook a cross-sectional electronic survey in six junior and senior high schools in Shanghai, China from late June to early July 2020. We evaluated the changes of sleep duration on weekdays by comparing sleep duration hours and insufficient sleep (< 9 h for children aged 6–13 years or < 8 h for teenagers aged 14–17 years) in COVID-19 school closures and after school reopening. We also investigated possible sex differences in the changes of sleep duration.

**Results:**

A total of 3265 students completed the survey, the mean age was 14.56 ± 1.99 years, 1567 (47.99%) were girls and 1344 (41.17%) were in grades 10–12. The overall sleep duration decreased from 8.88 h in school closures to 7.77 h after school reopening, and the change (difference: − 1.11 h; 95%CI: − 1.16, − 1.07; *P* < 0.001) was statistically significant. The prevalence of insufficient sleep increased sharply from 21.10 to 63.98%, and the change (ratio:3.03; 95%CI:2.84, 3.23; *P* < 0.001) was statistically significant. Besides, the changes were greater in girls than in boys.

**Conclusion:**

Results of this study revealed that sleep duration was longer and percentage of sufficient sleep was higher during COVID-19 school closures in adolescent students.

## Introduction

Sufficient sleep during adolescence is particularly important for not only rapid biologic growth and development, but also mental and emotional health [1, 2]. However, adolescents frequently report insufficient sleep [2]. Self-reported sleep duration in children and adolescents has decreased over the last 20 years [3]. Insufficient sleep could lead to a host of adverse health and social outcomes, including poorer school performance, psychiatric disorders, obesity, diabetes, and cardiovascular disease [4]. Insufficient sleep in adolescent students is highly prevalent and persists globally [5], with over 77% of the Chinese Han students aged 9–18 years reporting insufficient sleep in the 2014 Chinese National Survey on Students Constitution and Health [6].

The coronavirus disease 2019 (COVID-19) pandemic is an unprecedented international public health emergency affecting 216 countries, areas or territories around the world [7]. To contain the spread of COVID-19, stay at home orders, the banning of groups congregating, mandating work, social and educational venue closures have all proven to be successful non-pharmaceutical intervention adopted by authorities [8], impacting nearly 1.6 billion learners (more than 90% of the world’s students) [9]. The COVID-19 induced stay-at-home orders and online learning practices have been changing every aspect of our daily lives around the world, including sleep. While a few studies have reported the effects of the COVID-19 lockdown on sleep patterns among adults and college students [10, 11], it remains unclear how the school closures during COVID-19 pandemic affects adolescent sleep.

In China, the first confirmed human case of COVID-19 was reported in December 2019 in Wuhan City. As the first epicenter of COVID-19, Wuhan City was locked down starting Jan 23 to prevent further spread of the disease and the travel restrictions were lifted on April 8, 2020 [12]. Shanghai launched its highest-level emergency response against COVID-19 on Jan 24, 2020. The schools remained closed and online courses were offered in March. Shanghai schools reopened starting from April 27 and all school-age children returned to schools by June 2, 2020 [13]. The online courses closed at the end of the semester. As of August 11, Shanghai had reported 798 confirmed COVID-19 cases, with 7 deaths [14].

Given the above, we aimed to investigate the impact of COVID-19 induced lockdown and conversion to remote learning on sleep duration with a sample of adolescent students in Shanghai, China. We hypothesized that adolescents would report sleeping longer during the lockdown because they didn’t need to wake early in the morning to travel to school on weekdays or attend the pervasive after-school classes as usual.

## Methods

### Study design and participants

We used an uncontrolled before-after study design to assess sleep duration currently (after school reopening) and retrospectively (during school closures) at a single time point between Jun 28 and Jul 13, 2020. We undertook a cross-sectional online survey in two junior high schools and four combined junior and senior high schools in Jing’an District, Shanghai, China. We recruited all the students (*n* = 3564) before the survey, and 3265 participants aged 10 to 20 years agreed to participate, resulting in a response rate of 91.61%.

Our electronic questionnaire was powered by Tencent Questionnaire (https://wj.qq.com). The data was were collected online anonymously through a uniform resource locator, which was distributed to all participants via WeChat with the help of coordinators in the six schools and local education authorities. The final dataset was exported to Stata/SE 15.1 (StataCorp LLC, College Station, TX, USA) for the data analyses.

### Ethical approval and consent to participate

During the ongoing Covid-19 pandemic, we obtained the consent remotely using digital method. A week or so before the survey, we sent out passive consent letters to the parents or guardians of all subjects via their schools. The parents or guardians were required to return an electronically signed form only if they did not want their child to participate in the survey. We obtained active consent from the subjects by asking all subjects if they agreed to participate at the very beginning of the online survey.

The study was reviewed and approved by the institutional ethics board of Shanghai Institute of Planned Parenthood Research, and follows the ethical principles of the Declaration of Helsinki 1964.

### Measures

We measured sleep duration by collecting the bedtime and wake times on weekdays using four items that asked, “When do you usually go to bed?” and “When do you usually wake up?” on weekdays during the lockdown and after school reopening.

Bedtimes ranged from “No later than 18:00” to “1:00 or later” in half-hour intervals, and wake times ranged from “No later than 5:00” to “8:00 or later” with a 30-min interval. When calculating sleep duration, we used the time at the midpoint for each 30-min interval (e.g., 23:15 for “23:01 ~ 23:30”) and the maximum or minimum time at the ends of the two scales (e.g., 5:00 for “No later than 5:00” and 8:00 for “8:00 or later”) [15]. We also generated insufficient sleep variable if the participants slept less than the age group recommended hours per day according to National Sleep Foundation (e.g., ≥9 h for school-aged children aged 6–13 years and ≥ 8 h for teenagers aged 14–17 years) [16].

We asked the participants about their parental marital status using the question “As of today, what is the marital status of your parents?” and defined family structure as nuclear family if their parents were married, or other types if their parents were separated, divorced or widowed. We collected the highest level of education attained by the question “What’s the highest level of schooling attained for your father and mother respectively?”, and defined parental education as the higher level of the maternal and paternal educational attainment, and dichotomized it into “higher school or below” and “college or above”. We defined participants as only child if they reported having zero siblings. We asked the participants “How concerned are you about not being able to finish your current school grade or pass to the next grade?” , and labeled the participants as concerned if they were very concerned or somewhat concerned. We asked the participants “Do you know anyone personally (friends, family, or neighbors) who has had the COVID-19, including yourself?” and labeled the participants as being severely affected by COVID-19 if they answered “Yes”.

### Statistical analysis

All analyses were run separately for each sex with Stata/SE 15.1 because existing studies suggest that adolescent girls have a shorter weekday sleep duration than boys [17, 18]. We first analyzed the sample characteristics using chi-square statistics. We then estimated hours of sleep durations and prevalence of insufficient sleep with 95% confidence intervals (CIs) during school closures and after reopening across grades. Differences and ratios between school closures and reopening were tested using paired t-test and mcc command, respectively. Considering that these data are hierarchical, with participants within grades nested within schools, we examined the effects of potential confounders on the quantitative and qualitative changes in sleep duration (hours and from sufficient sleep to insufficient sleep) between school closures and after reopening using a multilevel generalized linear model and multilevel logistic regression model respectively. A two-sided *p*-value of 0.05 or less is considered statistically significant.

## Results

A total of 3265 students completed the survey. As summarized in Table 1, the mean age was 14.56 ± 1.99 years, 1567 (47.99%) were girls and 1344 (41.17%) were in grades 10–12. The parents of girls had higher educational attainment than those of boys (79.13% vs. 75.88%, *p* = 0.029). More than 70% of the participants had no siblings. About 44% were concerned about finishing the current school grade. And 1.16% were severely affected by COVID-19.

**Table 1:** Descriptive statistics of sample demographic characteristics

	Boys (*n* = 1698)	Girls (*n* = 1567)	Total (*n* = 3265)
Age (years)	14.56(2.01)	14.56(1.97)	14.56(1.99)
Grade			
6th	309(18.20)	268(17.10) ^*^	577(17.67)
7th	256(15.08)	263(16.78)	519(15.90)
8th	256(15.08)	210(13.40)	466(14.27)
9th	170(10.01)	189(12.06)	359(11.00)
10th	350(20.61)	311(19.85)	661(20.25)
11th	306(18.02)	256(16.34)	562(17.21)
12th	51(3.00)	70(4.47)	121(3.71)
Family structure			
Nuclear family	1498(88.22)	1381(88.13)	2879(88.18)
Other types	200(11.78)	186(11.87)	386(11.82)
Parental education			
High school or below	390(24.12)	318(20.87) ^*^	708(22.54)
College or above	1227(75.88)	1206(79.13)	2433(77.46)
Only Child			
Yes	1226(72.20)	1152(73.52)	2378(72.83)
No	472(27.80)	415(26.48)	887(27.17)
Adolescents severely affected by COVID-19			
Yes	24(1.41)	14(0.89)	38(1.16)
No	1674(98.59)	1553(99.11)	3227(98.84)
Grade concerned			
Concerned	688(43.57)	677(45.77)	1365(44.64)
Not concerned	891(56.43)	802(54.23)	1693(55.36)

Note: Data are n (%) or mean (SD). *P < 0.05 for the sex differences

The sleep duration (hours) in COVID-19 school closures and after school reopening, and the corresponding changes were computed overall and by grade (6th - 12th) and sex (boys or girls) in Table 2. The overall sleep duration was 8.88 h during school closure and 7.77 h once schools reopened. The change was statistically significant (t = − 51.18, *p* < 0.001). The findings remained robust across sex and grades.

**Table 2:** The hours of sleep duration in school closure and after reopening

	Boys mean (95% CI)	Girls mean (95% CI)	Total mean (95% CI)
Grade 6			
School lockdown	9.49(9.38,9.60)	9.18(9.06,9.30)	8.88(8.84,8.92)
School reopen	8.61(8.51,8.72)	8.42(8.31,8.53)	7.77(7.72,7.81)
Difference	−0.87(− 1.00, − 0.75) ^***^	−0.76(− 0.89, − 0.64) ^***^	−0.82(− 0.91, − 0.73) ^***^
Grade 7			
School lockdown	9.36(9.23,9.50)	9.17(9.05,9.28)	9.26(9.18,9.35)
School reopen	8.60(8.45,8.69)	8.21(8.11,8.32)	8.39(8.31,8.47)
Difference	−0.79(− 0.92, − 0.67) ^***^	−0.95(− 1.06, − 0.85) ^***^	−0.87(− 0.95, − 0.79) ^***^
Grade 8			
School lockdown	8.79(8.66,8.93)	8.71(8.58,8.84)	8.75(8.66,8.85)
School reopen	7.90(7.77,8.04)	7.58(7.46,7.70)	7.76(7.66,7.85)
Difference^#^	−0.89(−1.02, − 0.76) ^***^	−1.13(− 1.24, − 1.01) ^***^	−1.00(− 1.09, − 0.91) ^***^
Grade 9			
School lockdown	8.65(8.49,8.82)	8.83(8.67,8.99)	8.75(8.63,8.86)
School reopen	7.85(7.66,8.03)	7.61(7.49,7.74)	7.72(7.61,7.83)
Difference^#^	−0.81(−1.00, − 0.61) ^***^	−1.22(− 1.37, − 1.07) ^***^	−1.02(1.15, 0.90-) ^***^
Grade 10			
School lockdown	8.69(8.54,8.83)	8.71(8.57,8.86)	8.70(8.60,8.80)
School reopen	7.31(7.14,7.47)	7.01(6.88,7.15)	7.17(7.06,7.28)
Difference^#^	−1.38(− 1.55, − 1.21) ^***^	−1.70(− 1.85, − 1.55)	−1.53(− 1.64, − 1.42) ^***^
Grade 11			
School lockdown	8.69(8.52,8.86)	8.53(8.37,8.68)	8.62(8.50,8.73)
School reopen	7.64(7.47,7.82)	7.04(6.91,7.17)	7.37(7.26,7.48)
Difference^###^	−1.05(− 1.21, −0.88) ^***^	− 1.49(− 1.62, 1.35) ^***^	−1.25(− 1.36, − 1.13) ^***^
Grade 12			
School lockdown	8.20(7.84,8.55)	8.16(7.87,8.46)	8.18(7.95,8.40)
School reopen	7.07(6.63,7.51)	6.69(6.34,7.03)	6.85(6.58,7.12)
Difference	−1.13(− 1.56, −0.69) ^***^	− 1.48(− 1.76, − 1.19) ^***^	−1.33(− 1.57, − 1.08) ^***^
All Grades			
School lockdown	8.93(8.87,8.99)	8.83(8.77,8.88)	8.88(8.84,8.92)
School reopen	7.93(7.87,8.00)	7.59(7.53,7.65)	7.77(7.72,7.81)
Difference^###^	−1.00(−1.06, −0.94) ^***^	− 1.23(− 1.29, − 1.18) ^***^	−1.11(− 1.16, − 1.07) ^***^

Note: ^***^ *P* < 0.001 for the differences between school lockdown and reopen. ^#^*P* < 0.05 for the sex differences. ^###^*P* < 0.001 for the sex differences

Similar to Table 2, we estimated the prevalence of insufficient sleep before and after school reopening (Table 3). The prevalence of insufficient sleep increased significantly from 13.93 to 24.96% during school closures, and from 52.65 to 71.86% after school reopening. There is about a 3 to 4-fold increase in insufficient sleep after school reopening.

**Table 3:** The prevalence of sleep insufficient in school closure and after reopening

	Boys % (95% CI)	Girls % (95% CI)	Total % (95% CI)
Grade 6			
School lockdown	18.77(14.42,23.12)	28.36(22.96,33.75)	23.22(19.78,26.67)
School reopen	58.58(53.08,64.07)	71.64(66.25,77.04)	64.64(60.74,68.55)
Ratio	3.12(2.49,3.90) ^***^	2.53(2.11,3.03) ^***^	2.78(2.41,3.21) ^***^
Grade 7			
School lockdown	21.48(16.45,26.52)	28.14(22.70,33.57)	24.86(21.14,28.57)
School reopen	61.72(55.76,67.67)	76.43(71.30,81.56)	69.17(65.20,73.14)
Ratio	2.87(2.29,3.61) ^***^	2.72(2.25,3.27) ^***^	2.78(2.41,3.22) ^***^
Grade 8			
School lockdown	18.75(13.97,23.53)	15.71(10.79,20.64)	17.38(13.94,20.82)
School reopen	50.39(44.27,56.52)	63.33(56.82,69.85)	56.22(51.72,60.73)
Ratio	2.69(2.11,3.43) ^***^	4.03(2.99,5.43) ^***^	3.23(2.68,3.91) ^***^
Grade 9			
School lockdown	12.35(07.41,17.30)	15.34(10.21,20.48)	13.93(10.35,17.51)
School reopen	48.24(40.74,55.75)	56.61(49.55,63.68)	52.65(47.48,57.81)
Ratio	3.90(2.61,5.85) ^***^	3.69(2.67,5.09) ^***^	3.78(2.94,4.87) ^***^
Grade 10			
School lockdown	22.29(17.93,26.65) ^***^	21.22(16.68,25.77) ^***^	21.79(18.64,24.93)
School reopen	68.00(63.11,72.89)	76.21(71.47,80.94)	71.86(68.43,75.29)
Ratio	3.05(2.51,3.72) ^***^	3.59(2.912,4.43) ^***^	3.30(2.86,3.81) ^***^
Grade 11			
School lockdown	23.53(18.78,28.28)	24.22(18.97,29.47)	23.84(20.32,27.37)
School reopen	57.52(51.98,63.05)	73.05(67.61,78.48)	64.59(60.64,68.54)
Ratio	2.44(2.02,2.96) ^***^	3.02(2.45,3.72) ^***^	2.71(2.35,3.12) ^***^
Grade 12			
School lockdown	13.73(4.28,23.17)	14.29(6.09,22.48)	14.05(7.86,20.24)
School reopen	47.06(33.36,60.76)	62.86(51.54,74.18)	56.20(47.36,65.04)
Ratio	3.43(1.71,6.86) ^***^	4.40(2.55,7.59)	4.00(2.61,6.13) ^***^
All Grades			
School lockdown	19.96(55.83,60.53)	22.34(20.27,24.40)	21.10(19.74,22.54)
School reopen	58.19(55.84,60.53)	70.26(68.00,72.52)	63.98(62.32,65.61)
Ratio	2.91(2.66,3.20) ^***^	3.15(2.88,3.44) ^***^	3.03 (2.84,3.23) ^***^

Note: ***P < 0.001

We found few confounders were associated with changes in sleep duration as shown in Tables 4 and 5. Girls appeared to get on average 0.22 h less sleep than boys, and only child was associated with larger decrease (0.16 h) in sleep duration among boys only. Compared with boys, girls were at higher risk of switching the sleep pattern from sufficient sleep during school closure to insufficient sleep after school reopening (odds ratio = 1.33, 95%CI = 1.16,1.54).

**Table 4:** Association between changes in sleep duration and potential confounders

	Boys Coefficient (95% CI)	Girls Coefficient (95% CI)	Total Coefficient (95% CI)
Sex			
Girls	NA	NA	−0.22 (− 0.31, − 0.14) ^***^
Family structure			
Other types	0.18(− 0.02,0.38)	0.52(− 0.12,0.22)	0.11(− 0.02,0.24)
Parental education			
College or above	−0.02(− 0.18,0.14)	0.04(− 0.09,1.03)	−0.01(− 0.12,0.09)
Only Child			
Yes	−0.16(− 0.30, − 0.01) ^*^	−0.04(− 0.16,0.09)	−0.11(− 0.21, − 0.01)
Adolescents severely affected by COVID-19			
Yes	0.56(− 0.02,1.14)	0.47(− 0.09,1.03)	0.55(0.15,0.95)
Grade concerned			
Concerned	0.04(−0.09,0.17)	−0.02(− 0.13,0.10)	0.01(− 0.07,0.10)

Note: ^*^ *P* < 0.05, ^***^ *P* < 0.001, NA = Not applicable

**Table 5:** Association between changes in insufficient sleep and potential confounders

	Boys Odds ratio (95% CI)	Girls Odds ratio (95% CI)	Total Odds ratio (95% CI)
Sex			
Girls	NA	NA	1.33(1.16,1.54) ^***^
Family structure			
Other types	0.98(0.77,1.25)	0.95(0.68,1.32)	0.97(0.77,1.22)
Parental education			
College or above	1.16(0.90,1.51)	1.24(0.95,1.63)	1.19(0.99,1.44)
Only Child			
Yes	0.98(0.77,1.25)	1.09(0.85–1.39)	1.02(0.86,1.22)
Adolescents severely affected by COVID-19			
Yes	0.53(0.19,1.51)	0.40(0.12,1.29)	0.46(0.21,1.02)
Grade concerned			
Concerned	0.96(0.79,1.19)	1.02(0.82,1.27)	0.98(0.84,1.15)

Note: ^***^ P < 0.001, NA = Not applicable

## Discussion

In summary, we found that adolescent sleep duration was longer during COVID-19 school closures. The reported sleep duration during COVID-19 school closures was one hour longer compared to after school reopening, with more than an hour for senior high school students and less than an hour for junior high school students. The prevalence of insufficient sleep almost tripled after school reopening, with a nearly fourfold increase for ninth-grade students.

The quantitative research on the effect of COVID-19 shutdown on sleep duration in adolescent students mixed. The prevalence of insufficient sleep per night for weekdays reported in a study decreased from 16% during COVID-19 Stay-at-Home orders to 8% in college students [10]. A cross-sectional survey across different continents found that adolescents had fourfold increased odds for longer sleep duration on weekdays during the pandemic [19]. Another longitudinal study in the United States revealed that the percentage of sufficient sleep in school night increased to 64% during COVID-19 pamdemic from 57% before COVID-19 in adolescents, and this change was statistically non-significant. The main disadvantage of the above mentioned studies are the small sample size. Using a large sample of adolescent students, our study suggested that adolescent slept better during COVID-19 school closures in Shanghai, measured by sleep duration or percentage of sufficient sleep.

Longer sleep duration in adults could be attributed to decreased work-related activities but not leisure activities during the COVID-19 lockdown [11]. For school students, COVID-19 lockdown closed the schools and shifted the traditional classes in school to online courses. Online learning eliminates travel time to school, suggesting that school commuting time may reduce adolescent sleep [20]. Early school start times serve as a key modifiable contributor to insufficient sleep after school reopening. The American Academy of Sleep Medicine recommends middle and high schools should start at 8:30 AM or later [21]. The Ministry of Education and Shanghai municipal education committee both suggest middle schools and high schools start at 8:00 AM and 7:45 AM respectively [22]. However, many schools in China don’t follow the recommendations of education authorities as expected, and bring forward the school start time. For example, according to the timetable on one Shanghai high schools’ website, the students need to wake up at 6:15 AM on weekdays [23].

In addition to eliminating the commuting time, lower academic stress is another potential reason for improved sleep duration during COVID-19 school closures, although we didn’t measure it in the present study. Chinese society attaches great importance to education, and Confucianism links an individual’s academic success to family honor [24], which places school students under tremendous pressure. It appears that the learning load was reduced during COVID-19 pandemic, because the National Health Commission of the People’s Republic of China suggests that all middle and high school students in China should take no more than four hours of online learning per day [25].

The third possible reason is that all the offline private educational institutions were also closed in response to COVID-19 pandemic. While it is uncommon in Western culture, attending night school for extra study is a very common practice in Asian countries [24], including the communities the participants lived in although we didn’t collect information on attending night schools. The supplemental tutoring that many students receive was closed during COVID-19 school closures may also contribute to longer sleep duration in this period.

Consistent with existing studies [18, 26], our findings suggested that adolescent girls slept less on weekdays than boys. Compared with boys, girls were more at risk of insufficient sleep after school reopening. The social requirements, including morning grooming [26], may in part explain the gender differences in sleep duration on weekdays.

### Limitation

Caution is needed when interpreting our results. The primary limitation is the cross-sectional design of the study precludes assumptions of causality and further longitudinal studies are needed to confirm these findings. Second, it is important to note that self-reported sleep duration in our study overestimated actual sleep duration when objectively measured [27], because bedtime was measured, and sleep onset latency and night wakings were not collected in our study. However, given that we used the same method to collect the bedtime and wake times both retrospectively and currently at a single time point, the changes in sleep duration are most likely to be valid despite imprecisions. Third, the responses of bed and wake times are limited range. If the reported times are at the ends of the scale, the sleep duration may be over or underestimated, although the prevelance may be relatively low. Fourth, given that the COVID-19 pandemic is unpredictable, the study didn’t measure the sleep duration during school breaks to allow us to disentangle the unique disruptions of the COVID-19 pandemic. Last, we are unable to assess whether our findings are generalizable beyond Shanghai, more studies in different settings are needed.

## Conclusions

The adolescents reported obtaining shorter sleep duration after school reopening compared to during COVID-19 school closures. Early school start time may be the key potential modifiable factor that contributes to the reduced sleep duration [28, 29].

## Data Availability

The datasets used and/or analyzed during the current study are available from the corresponding authors on reasonable request.

## Abbreviations

COVID-19: Coronavirus disease 2019
CI: confidence interval
